# The Impact of Efflux Pump Inhibitors on the Activity of Selected Non-Antibiotic Medicinal Products against Gram-Negative Bacteria

**DOI:** 10.3390/molecules22010114

**Published:** 2017-01-11

**Authors:** Agnieszka E. Laudy, Ewa Kulińska, Stefan Tyski

**Affiliations:** 1 Department of Pharmaceutical Microbiology, Medical University of Warsaw, Oczki 3 Str., 02-007 Warsaw, Poland; ewa_kulinska@vp.pl (E.K.); s.tyski@nil.gov.pl (S.T.); 2 Department of Antibiotics and Microbiology, National Medicines Institute, Chełmska 30/34 Str., 00-725 Warsaw, Poland

**Keywords:** efflux, efflux pump inhibitor, non-antibiotics, medicinal products, Gram-negative bacteria, MDR, PAβN, drugs, quinolones

## Abstract

The potential role of non-antibiotic medicinal products in the treatment of multidrug-resistant Gram-negative bacteria has recently been investigated. It is highly likely that the presence of efflux pumps may be one of the reasons for the weak activity of non-antibiotics, as in the case of some non-steroidal anti-inflammatory drugs (NSAIDs), against Gram-negative rods. The activity of eight drugs of potential non-antibiotic activity, active substance standards, and relevant medicinal products were analysed with and without of efflux pump inhibitors against 180 strains of five Gram-negative rod species by minimum inhibitory concentration (MIC) value determination in the presence of 1 mM MgSO_4_. Furthermore, the influence of non-antibiotics on the susceptibility of clinical strains to quinolones with or without PAβN (Phe-Arg-β-naphthylamide) was investigated. The impacts of PAβN on the susceptibility of bacteria to non-antibiotics suggests that amitriptyline, alendronate, nicergoline, and ticlopidine are substrates of efflux pumps in Gram-negative rods. Amitriptyline/Amitriptylinum showed the highest direct antibacterial activity, with MICs ranging 100–800 mg/L against all studied species. Significant decreases in the MIC values of other active substances (acyclovir, atorvastatin, and famotidine) tested with pump inhibitors were not observed. The investigated non-antibiotic medicinal products did not alter the MICs of quinolones in the absence and in the presence of PAβN to the studied clinical strains of five groups of species.

## 1. Introduction

One of the most serious healthcare problems today is the increasing prevalence of antibiotic-resistant Gram-negative bacterial strains. The coexistence and cooperation of different drug-resistance mechanisms (e.g., the production of enzymes that destroy antibiotics and overexpression of the multidrug (MDR) efflux pumps that remove drugs from the cells of clinical bacterial isolates) make infectious diseases treatment difficult and ineffective [1,2,3]. In contrast to Gram-positive cocci, in Gram-negative rods, MDR efflux pumps from the resistance-nodulation-division (RND) family are present. Very important and dangerous for infected people is the phenomenon of removal of antibiotics belonging to different chemical groups from the bacteria by RND efflux systems [2,3].

Currently, there is an urgent need for both the discovery of new chemical compounds with potent and broad antibacterial activity and a search for methods enabling the restoration of bacterial susceptibility to known antibacterial agents (e.g., combination of β-lactamase inhibitor and β-lactam antibiotic).

Alternatively, looking for effective antibacterial agents among known medicinal products from the group of “non-antibiotics”, drugs belonging to different therapeutic groups that are used in the management of pathological symptoms of a non-infectious aetiology, is a particularly interesting strategy [4,5]. The group of “non-antibiotic” drugs may be divided into two subgroups, only one of which possesses direct antimicrobial activity. The subgroup not possessing direct activity consists of two subclasses: the first called “helper compounds” (they alter the permeability of bacteria to conventional antibiotics) and the second called “macrophage modulators” (they enhance the cytotoxic activity of macrophages that phagocytose bacteria) [4]. The potential role of non-antibiotics in the treatment of multidrug-resistant Gram-negative bacteria has recently been investigated. The antibacterial activity of several non-steroidal anti-inflammatory drugs (NSAIDs) has been demonstrated, such as that of diclofenac against *Escherichia coli* [6,7,8], *Klebsiella* sp. [6,7], *Proteus mirabilis*, *Pseudomonas aeruginosa*, *Stenotrophomonas maltophilia*, and *Acinetobacter baumannii*, as well as that of ibuprofen and naproxen against *S. maltophilia* [6]. The antibacterial activity of therapeutics belonging to other drug groups was also described: cardiovascular drugs (amlodipine and nifedipine) against *E. coli*, *Klebsiella* sp., and *Salmonella* sp. [9]; local anaesthetics (e.g., lignocaine, bupivacaine, ropivacaine, and tramadol) against *E. coli* [10,11,12] and *P. aeruginosa* [12]; vasoconstrictor drugs (e.g., oxymetazoline against *E. coli* [13]), and proton pump inhibitors (e.g., rabeprazole and lansoprazole) against *Helicobacter pylori* [14,15].

Moreover, it seems that the most important feature of non-antibiotic drugs, besides their therapeutic use, is their ability to inhibit or enhance the activities of some efflux pumps in Gram-negative rods. It is known that reserpine, used in the past as an antipsychotic and antihypertensive drug, inhibits efflux pumps in Gram-negative bacteria [16]. The reverse phenomenon was observed in the case of salicylate, a natural substrate for efflux pumps in *Burkholderia cenocepacia*, which can induce efflux-mediated resistance [17]. Salicylate-induced efflux pump expression has also been observed in *E. coli* [18], *Salmonella enterica* serovar Typhimurium [19], and *Campylobacter jejuni* [20].

It is worthy of note that in our previous studies, it was proven for the first time that active substances of NSAIDs (such as mefenamic acid, diclofenac, ibuprofen, naproxen, and acetylsalicylic acid) and relevant NSAID medicinal products (Mefacit, Olfen, Diclac, Nurofen, Naproxen, and Aspirin) are substrates for the efflux pumps present in Enterobacteriaceae as well as in non-fermentative Gram-negative rods [6]. 

Relying on our previous studies on the direct antibacterial activity of non-antibiotics [13,21,22,23,24] and on our research concerning the active removal of NSAIDs by efflux pumps [6], we decided to investigate whether non-antibiotic drugs from the other therapeutic groups may also be substrates for MDR efflux pumps in Gram-negative rods. It is highly likely that the presence of efflux pumps may be one of the reasons for the weak activity of non-antibiotics—as was observed in the case of some NSAIDs—against Gram-negative rods. Such a study would shed new light on the participation of MDR efflux pumps in the drug resistance of Gram-negative rods. The aim of this study is also to explain whether the non-antibiotic drugs that are efflux pump substrates could modify efflux-related bacterial resistance to antibiotics.

The research was performed in two steps: (i) determination of the susceptibility of standard and clinical Gram-negative strains to selected quinolones and non-antibiotics from different groups (both active substances and medicinal products) tested in the presence or absence of efflux pump inhibitors (EPIs); (ii) investigation of the influence of non-antibiotics (which probably are extruded by efflux pumps) with or without EPIs on the susceptibility of clinical strains to quinolones.

## 2. Results

### 2.1. Susceptibility of Bacteria to Non-Antibiotic Active Substances and Medicinal Products

While determining the susceptibility of all of the investigated bacterial strains to potential non-antibiotics and active substances as well as to the relevant medicinal products, a dilution difference of only 2-fold between the minimum inhibitory concentration (MIC) values of active substances present in standard or tablet form was seldom observed. The content of the active compounds was considered and calculated. The information concerning the antibacterial activity of the preparations assessed in the study is shown in Table 1. Among the analysed material, only the medicinal products containing one of the three standard substances—such as alendronate sodium, amitriptyline, and carboplatin—were active (MIC ≤ 800 mg/L) against some of the tested standard strains of Gram-negative rods. Amitriptyline and its relevant medicinal product, Amitriptylinum tablets, showed the highest antibacterial activity, with MICs of 100–800 mg/L, as well as being active against all of tested standard strains. The remaining substances tested (i.e., acyclovir, atorvastatin, famotidine, nicergoline, and ticlopidine) did not inhibit the growth of the standard Gram-negative strains (MIC > 800 mg/L).

Three tested active substances (alendronate sodium, amitriptyline, and carboplatin) and relevant medicinal products were selected for further investigation of non-antibiotic activity against clinical strains. The characteristics of the susceptibility of 180 selected clinical strains belonging to five Gram-negative rod species to quinolones—such as nalidixic acid and ofloxacin, which are substrates of MDR efflux pumps—are presented in Table 2. A significant decrease in the MIC value of at least one of the tested quinolones was observed in the presence of PaβN (Phe-Arg-β-naphthylamide) in all of the clinical isolates used in this study.

There was one important observation: that amitriptyline and the relevant medicinal product (Amitriptylinum) were active against all 180 studied clinical strains, with MIC values ranging from 100 to 800 mg/L (Table 3). Moreover, 100% of clinical strains of *P. aeruginosa* and *S. maltophilia* were also sensitive to alendronate sodium (Ostenil), the MIC value of alendronate was ≤200 mg/L in the case of 92% and 28% bacterial strains, respectively. Furthermore, carboplatin was active only against clinical strains of *P. aeruginosa* (Table 3).

### 2.2. The Effect of EPIs on the Susceptibility of Bacteria to Drug Active Substances and Medicinal Products 

The susceptibility of 13 standard strains to eight drug active substances and relevant medicinal products in the presence of two efflux pump inhibitors was tested. For the study carried out on clinical strains, five active substances (amitriptyline, alendronate sodium, carboplatin, nicergoline, and ticlopidine) and their relevant medicinal products (Amitriptylinum, Ostenil, Carbosin, Niglostin and Apo-Clodin) were selected. They showed increased activity (decreases in MIC values of at least a 4-fold) against standard strains in the presence of PAβN compared with in its absence. Neither reserpine nor PAβN (both 50 mg/L) inhibited the growth of any of the tested strains.

Between the two studied pump inhibitors, only PAβN affected the susceptibility of Gram-negative rods to the analysed medicinal products/active substances. There were no differences greater than 2-fold between the MIC values of the active substances present in the standard and those in the tablet, when tested in the presence of efflux pump inhibitors.

The effect of PAβN on the MIC values of potential non-antibiotics for standard and clinical strains is presented in Table 1 and Table 4, respectively. Among the tested active substances/medicinal products, the MIC values of alendronate/Ostenil, ticlopidine/Apo-Clodin, and amitriptyline/Amitriptylinum in the presence of PAβN were significantly (a minimum 4-fold) reduced against the majority of the standard strains (8 out of 13, 62% for the first two non-antibiotics, and 7 out 13, 54%, respectively) (Table 1). Additionally, an increase of at least 4-fold in bacterial susceptibility to nicergoline/Niglostin (for *E. coli*, *K. pneumoniae*, and *A. baumannii* standard strains) and to carboplatin/Carbosin (for *P. aeruginosa* ATCC 27853) was observed. Significant (a minimum 4-fold) decreases in the MIC values of other tested active substances (acyclovir, atorvastatin, and famotidine) with pump inhibitors were not obtained.

As shown in Table 4, ticlopidine/Apo-Clodin and amitriptyline/Amitriptylinum tablets were the two analysed active substances/medicinal products for which there were significant decreases in the MIC values in the presence of an inhibitor among the majority of strains belonging to 4 out of 5 studied species of Gram-negative rods. The highest increase in bacterial susceptibility to non-antibiotic in the presence of PAβN was observed in the case of ticlopidine/Apo-Clodin. The MIC values of ticlopidine/Apo-Clodin for all 180 studied strains were >800 mg/L, and decreased in the case of all isolates of *E. coli* (to 25 mg/L) and *K. pneumoniae* (to 25–100 mg/L), in 33 out of 36 strains of *S. maltophilia* (to 25–800 mg/L), and in 30 out of 36 strains of *A. baumannii* (to 25–400 mg/L). Additionally, a large decrease in the MIC values of nicergoline/Niglostin was noticed: MICs changed from >800 mg/L to 50–200 mg/L for *A. baumannii* (100% strains) and to 100–200 mg/L for *E. coli* (100%), respectively.

Moreover, the comparably high increase in the susceptibility of *E. coli* to alendronate/Ostenil (MICs changed from >800 mg/L to 100–400 mg/L) was obtained. In the case of amitriptyline/Amitriptylinum, in the presence of PAβN, the MIC values of these studied materials against all Gram-negative rods decreased from 200–800 mg/L to 25–400 mg/L.

### 2.3. The Effect of Non-Antibiotics on the Susceptibility of Clinical Strains to Quinolones with and without PAβN

The susceptibility of clinical strains to antimicrobial agents (using quinolones as an example) that are actively extruded by efflux pumps in the presence of non-antibiotics was studied. Quinolones for each group of species and non-antibiotic medicinal products were selected on the basis of data obtained in previous investigations (Table 2 and Table 4). A decrease of at least 4-fold in the MIC values of the selected medicinal products—including Ostenil, Apo-Clodin, Amitriptylinum, Niglostin, and Carbosin—was previously observed in the presence of PAβN (Table 4).

Among all 180 clinical strains used in this study, none of the investigated medicinal products affected the susceptibility of the studied clinical strains of five group of species in the absence and in the presence of PAβN.

## 3. Discussion

The potential use of non-antibiotics for the treatment of infections caused by resistant bacteria is a very interesting strategy. Unfortunately, the majority of non-antibiotic agents belonging to different therapeutic groups—such as anti-inflammatory drugs, antianaplastics, anticonvulsants, antiarrhythmics, antihypertensives, antidepressants, and spasmolytics—show only marginal direct antibacterial activity (MIC ≥ 3000 mg/L) [6,22,23]. Some of these agents (e.g., most phenothiazines [25], some anaesthetics [13], antihistamines [13], *trans*-chlorprothixene [5], and dodecyl(C(12))gallate(3,4,5-trihydroxybenzoate) [26]) are active only against Gram-positive cocci. Only in the case of a few non-antibiotics—some phenothiazines (promazine [27] and chloropromazine [5]), 2-dimethyl-amino-ethylchloride [27], oxymetazoline [13], and sertraline [21]—has significant activity been described against both Gram-positive as well as Gram-negative bacteria. Relying on our previous article concerning the influence of efflux pump inhibitors on the activity of non-antibiotic NSAIDs against Gram-negative rods [6], we have decided to investigate whether non-antibiotic drugs from the other therapeutic groups are also substrates for MDR efflux pumps of Gram-negative rods. Perhaps the presence of efflux pumps may be one of the reasons for the weak activity of non-antibiotics, as in the case of some NSAIDs [6], against Gram-negative rods. Such a possibility is also supported by the fact that MDR efflux pumps from the RND family are able to remove antibiotics of different chemical groups as well as some disinfecting agents, aromatic hydrocarbons, acriflavine, rhodamine 6G, vanadium, crystal violet, and ethidium bromide from bacterial cells [3].

In this study, we selected the following eight active substances from different classes of drugs: acyclovir (antivirals), alendronate (specific inhibitor of osteoclast-mediated bone resorption), amitriptyline (antidepressant), atorvastatin (a cholesterol-lowering drug), carboplatin (anticancer drug), famotidine (antihistamines), nicergoline (vasodilators used to treat senile dementia), and ticlopidine (antiplatelet drug). Drugs of these groups may be taken by the patient for a long time or permanently. Among eight analysed potential non-antibiotics (as active substances and relevant medicinal products), in the case of five of them (amitriptyline, alendronate sodium, carboplatin, nicergoline, and ticlopidine) a final 4-fold decrease in the MIC values of the agent in the presence of the efflux pump inhibitor, PAβN, was observed. This compound is the most commonly used MDR efflux pump inhibitor for a basic in vitro phenotypic screening test of antibiotic removal from bacteria by MDR efflux pumps [1,16,28,29,30,31,32,33,34]. This test is based on measuring the changes in the MICs of the antibiotic in the absence and presence of the efflux pump inhibitor [28,29,35,36]. It is known that PAβN potently inhibits the efflux systems from the Mex family in *P. aeruginosa* (especially MexAB-OprM) [3,29,34,37] as well as inhibits the AcrAB-TolC efflux system, the presence of which was described in the strains of the Enterobacteriaceae family species (e.g., *E. coli* [32,37,38], *Enterobacter aerogenes* [39], *Klebsiella pneumoniae* [40], *S. enterica* serovar Typhimurium [41], and *P. mirabilis* [42]).

In this study, it was shown for the first time that non-antibiotic active substances (such as amitriptyline, alendronate sodium, nicergoline, and ticlopidine) were actively removed, most probably by the efflux pumps present in Enterobacteriaceae as well as in non-fermentative Gram-negative rods. Interestingly, ticlopidine/Apo-Clodin and amitriptyline/Amitriptylinum were the two analysed active substances/medicinal products in this study that displayed a significant decrease in MIC in the presence of an efflux pump inhibitor in the case of 4 out of 5 studied species of Gram-negative rods.

Similarly, in our earlier article, we described the increased sensitivity of the majority of *P. aeruginosa*, *S. maltophilia*, *A. baumannii, E. coli*, *K. pneumoniae*, and *P. mirabilis* strains to four NSAIDs (diclofenac, ibuprofen, mefenamic acid, and naproxen) in the presence of PAβN [6]. It was described that salicylic acid may be a substrate for the CeoAB-OpcM efflux system in *B. cenocepacia* [17] and that acetylsalicylic acid (Aspirin) is probably a substrate for efflux pumps presented in some strains of *E. coli* and *P. mirabilis* [6].

A particularly important observation of the present study is the restoration of susceptibility of *E. coli*, *K. pneumoniae*, *S. maltophilia*, and *A. baumannii* clinical strains to ticlopidine in the presence of the efflux pump inhibitor. Recently, it has been proven that ticlopidine, not possessing antibacterial activity, displayed potential synergistic activity with cefuroxime against Gram-positive cocci [43]. Ticlopidine inhibits cell wall teichoic acid synthesis and, in this way, blocks the cooperative action of penicillin-binding proteins (PBPs), which in turn sensitises methicillin-resistant *Staphylococcus aureus* (MRSA) strains to β-lactams. Observation of the susceptibility of *E. coli*, *K. pneumoniae*, *S. maltophilia*, and *A. baumannii* to ticlopidine in the presence of PAβN indicates a different mechanism of action of this substance against Gram-negative rods, as compared with Gram-positive bacteria.

An equally important issue is the effect of non-antibiotic medicinal products on antibiotic treatment in the context of drug interactions with bacterial efflux pumps. Currently, analysing the non-antibiotic group, salicylic acid is the only known substrate of the MDR efflux pumps that can induce efflux-mediated resistance in some Gram-negative rods, such as *E. coli* [18], *S. enterica* serovar Typhimurium [19], and *B. cenocepacia* [17]. Moreover, in our previous article, we proved that one of the studied non-antibiotic medicinal products, Aspirin tablets (containing acetylsalicylic acid), reduced the antibiotic susceptibility of a few *E. coli* strains, but no clinical strains of *K. pneumoniae*, *P. mirabilis*, *P. aeruginosa*, *S. maltophilia*, or *A. baumannii* [6]. Also, salicylate did not affect the susceptibility level of *A. baumannii* to ciprofloxacin, gentamicin, or ceftriaxone [44].

The important observation from this study is that the impact of medicinal products containing alendronate, carboplatin, ticlopidine, nicergoline, and amitriptyline on the induction of resistance of Gram-negative rods to quinolones has not been demonstrated. Similarly, non-antibiotics that have been described as efflux pump system substrates (diclofenac, mefenamic acid, ibuprofen and naproxen) did not affect antibiotic resistance [6].

Quinolones in our previous and present studies were used as a model—an example of compounds which are actively removed by efflux pumps. The participation of the efflux pumps in fluoroquinolone resistance has been shown in a variety of Gram-negative rod genera [1,32,35]. However, keep in mind that the main mechanisms of resistance to fluoroquinolones are mutations in *gyr* and *par* genes [45].

Probably, non-antibiotic compounds that are efflux pump system substrates but do not affect antibiotic resistance (amitriptyline, alendronate, ticlopidine, and nicergoline) might be safely applied during antibacterial treatment.

## 4. Materials and Methods

### 4.1. Bacterial Strains and Growth Conditions

The following standard strains were used in the study: *E. coli* ATCC 25922, NCTC 10538 and NCTC 8196; *K. pneumoniae* ATCC 13883 and ATCC 700603; *Proteus vulgaris* ATCC 13315; *P. aeruginosa* ATCC 27853, NCTC 6749 and PAO1; *S. maltophilia* ATCC 13637 and ATCC 12714; *A. baumannii* ATCC 19606; and *Burkholderia cepacia* ATCC 25416. This study also included 180 clinical strains, i.e., 36 strains of 5 Gram-negative rod species (*E. coli*, *K. pneumoniae*, *P. aeruginosa*, *S. maltophilia*, and *A. baumannii*). Clinical strains were isolated from samples of different materials derived from hospitalized patients in Warsaw in the period 2007–2010 and were identified in hospital microbiological laboratories by routine microbiological methods using API tests (bioMérieux, Marcy l’Etoile, France). All strains were stored at −80 °C until analysis. Prior to testing, each strain was subcultured twice on TSA (bioMérieux) medium for 24–48 h at 30 °C to ensure viability.

### 4.2. Quinolones, Efflux Pump Inhibitors, Active Substances, and Medicinal Products of Drugs

The following eight drugs of potential non-antibiotic activity, active substance standards and nine relevant medicinal products were analysed in this study: acyclovir (Hasco-Lek, Wrocław, Poland) and Zovirax inj 25 mg/mL (Wellcome, London, UK), alendronate (Polpharma SA, Starogard Gdański, Poland) and Ostenil tab 70 mg (Teva Pharmaceuticals, Kraków, Poland), amitriptyline (ICN Polfa Rzeszów SA, Rzeszów, Poland) and Amitriptylinum tab 10 mg (ICN Polfa Rzeszów SA), atorvastatin (Teva Pharmaceuticals) and Atorvox tab 10 mg (Farmacom, Kraków, Poland), carboplatin (Heraeus, Hanau, Germany) and Carbosin inj 10 mg/mL (Pharmachemie BV, Haarlem, Netherlands), famotidine (Gedeon Richter, Vegyeszti Gyar Nyrt, Hungary) and Famogast tab 20 mg (Polpharma SA, Starogard Gdański, Poland), nicergoline (European Pharmacopeia, Strasbourg, France) and Niglostin tab 10 mg (Hasco-Lek, Wrocław, Poland), ticlopidine (Sanofi-Synthelabo, Mumbai, India) and Apo-Clodin tab 250 mg (Apotex, Leiden, The Netherlands).

**Figure 1:**
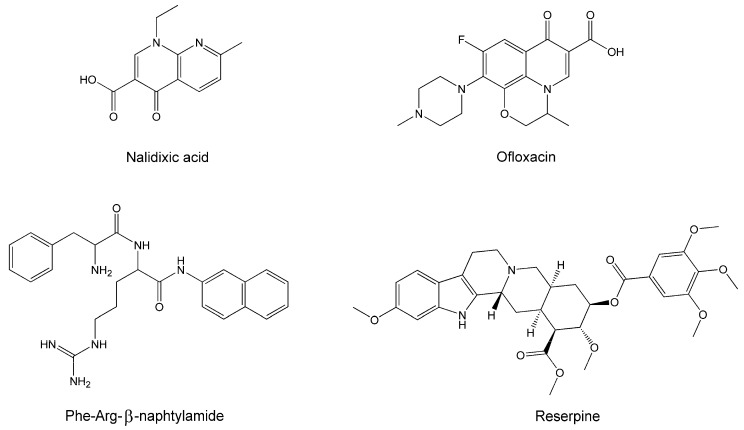
Quinolones and efflux pump inhibitors used in the study.

Two quinolones, nalidixic acid (Sigma, St. Louis, MO, USA) and ofloxacin (Sigma), as well as two efflux pump inhibitors, Phe-Arg-β-naphtylamide (PAβN) and reserpine, both from Sigma, were also used in this study (Figure 1).

### 4.3. Determination of the MICs of Quinolones, Medicinal Products/Active Substances of Drugs with and without an Efflux Pump Inhibitor

The MIC values of quinolones, as well as those of drugs, potential non-antibiotics, active substances and relevant medicinal products, in the presence or absence of EPIs, were estimated on Mueller–Hinton II (MH II) agar (Becton Dickinson, Franklin Lakes, NJ, USA) with 1 mM MgSO_4_ using double-agent dilutions according to the CLSI guidelines [46]. Bacterial suspensions at a density 0.5 McFarland units by Densimat (bioMérieux) were diluted 1:10 and 1.5 μL (10^4^ cfu/mL) and applied to the surface of the agar plates. The plates were incubated at 35 °C for 18 h. The assay was validated by MIC determination of selected quinolones against reference strains (*E. coli* ATCC 25922 and *P. aeruginosa* ATCC 27853) and the experimental values obtained were compared with the CLSI guidelines [47]. Moreover, the MIC values of medicinal products in the form of injections, such as the Zovirax inj and Carbosin inj, in the presence or absence of EPIs, were estimated not only on MH II agar but also in MH II broth (Becton Dickinson) with 1 mM MgSO_4_, using 2-fold serial dilutions, according to the CLSI guidelines [46] in order to compare these two assays.

To address the possibility that PAβN was permeabilizing the cells, antibiotic susceptibility assays were repeated in the presence of 1 mM MgSO_4_ to stabilize the outer membrane [48].

To estimate the MIC values of medicinal products, tablets were homogenized and then suspended in the same way as active substances. The amount of active substances in the material obtained was calculated in comparison with the concentrations of the appropriate drug active substances [6].

The MIC values of nalidixic acid, ofloxacin, drug active substances and medicinal products (tablets or solutions for injections), with or without pump inhibitors, PAβN or reserpine, were evaluated in order to determine the ability of strains to remove quinolones or non-antibiotics by MDR efflux pumps. The concentration of both EPIs applied was 50 mg/L. At decrease of at least 4-fold in the MIC values after the addition of PAβN or reserpine was considered significant [6].

### 4.4. Determination of Quinolone Activity in the Presence of Medicinal Products with and without PAβN

The influence of non-antibiotic medicinal products, which probably are removed by MDR efflux pumps, on the susceptibility of clinical strains from selected Gram-negative rod species to antimicrobial agents, in the presence or absence of PAβN, was investigated by determining the MIC values of quinolones, as described in our previous publication [6]. The MICs of antimicrobial agents were estimated on MH II agar medium with 1 mM MgSO_4_.

The selection of medicinal products utilised for this part of the study was based on the results obtained from the determination of the susceptibility of clinical strains to the potential non-antibiotics in the presence of efflux pump inhibitors. These medicinal products were selected when there was a minimum 4-fold decrease in the MIC values of the potential non-antibiotic in the presence of PAβN, when compared with the MIC values of the non-antibiotic alone. The following concentrations of particular medicinal products were equal to a quarter of the lowest MIC value determined for each group of species that were tested in the absence of PAβN: Ostenil (400 mg/L—*E. coli* and *K. pneumoniae*, 50 mg/L—*P. aeruginosa*), Apo-Clodin (200 mg/L—*E. coli*, *K. pneumoniae*, *A. baumannii*, and *S. maltophilia*), Amitriptylinum (200 mg/L—*P. aeruginosa*, 50 mg/L—*E. coli*, *K. pneumoniae*, and *A. baumannii*), Niglostin (200 mg/L—*E. coli*, *K. pneumoniae*, and *A. baumannii*), Carbosin (100 mg/L—*P. aeruginosa*), and in the presence of PAβN: Ostenil (100 mg/L—*K. pneumoniae,* 25 mg/L—*E. coli*, 6.25 mg/L—*P. aeruginosa*), Apo-Clodin (12.5 mg/L—*S. maltophilia*, 6.25 mg/L—*E. coli*, *K. pneumoniae*, and *A. baumannii*), Amitriptylinum (12.5 mg/L—*E. coli*, *K. pneumoniae*, and *P. aeruginosa*, 6.25 mg/L—*A. baumannii*), Niglostin (25 mg/L—*E. coli*, *K. pneumoniae*, and *A. baumannii*) and Carbosin (25 mg/L—*P. aeruginosa*).

A minimum 4-fold change in the MIC value of one of the quinolones after the addition of a non-antibiotic medicinal product was considered significant. Additionally, the effect of a non-antibiotic on the susceptibility of clinical strains to quinolones in the presence of PAβN was analysed. A minimum 4-fold change in the MIC value of nalidixic acid or ofloxacin in the presence of both non-antibiotic and PAβN, when compared with the MIC of quinolone in the presence of PAβN only, was considered relevant.

## 5. Conclusions

It was shown for the first time that at least 4-fold decrease in the MIC values of the non-antibiotic active substances (such as amitriptyline, alendronate, ticlopidine, and nicergoline) and relevant medicinal products (Amitriptylinum, Ostenil, Apo-Clodin, and Niglostin) was observed after the addition of PAβN. The data suggest that these non-antibiotics are substrates for efflux pump systems in some Gram-negative rods. The important observation is that none of the investigated non-antibiotic medicinal products containing amitriptyline, alendronate, ticlopidine, and nicergoline affect the antibiotic resistance of the strains. Moreover, amitriptyline/Amitriptylinum showed direct antimicrobial activity against standard strains and clinical isolates from all tested species of Gram-negative rods, while the two other non-antibiotics (alendronate and ticlopidine) were only active against some *S. maltophilia, P. aeruginosa*, and *E. coli* strains.

## Figures and Tables

**Table 1:** The activity of non-antibiotics (active substances and medicinal products) with and without the efflux pump inhibitor PAβN against standard Gram-negative strains.

Strains	MIC (mg/L)				
	alen/Ostenil ^a^ (+PAβN) ^b^	tic/Apo-Clodin ^a^ (+PAβN)^b^	amit/Amitriptylinum ^a^ (+PAβN)^b^	nic/Niglostin ^a^ (+PAβN)^b^	crb/Carbosin ^a^ (+PAβN)^b^
*E. coli* ATCC 25922	>800 **(200/100)**	>800 **(25)**	200 **(50)**	>800 **(100)**	>800 (>800)
*E. coli* NCTC 8196	>800 **(200/100)**	>800 **(25)**	200 **(50)**	>800 **(100)**	>800 (>800)
*E. coli* NCTC 10538	>800 **(200/100)**	>800 **(25)**	400 **(25)**	>800 **(100)**	>800 (>800)
*K. pneumoniae* ATCC 13883	>800 **(200)**	>800 **(25)**	200 **(50)**	>800 **(200)**	>800 (>800)
*K. pneumoniae* ATCC 700603	>800 (800)	>800 **(100)**	400 **(100)**	>800 (>800)	>800 (>800)
*P. vulgaris* ATCC 13315	>800 **(400)**	>800 (>800)	800 (800)	>800 (>800)	>800 (>800)
*P. aeruginosa* ATCC 27853	200 **(25)**	>800 (>800)	800 **(25)**	>800 (>800)	800 **(200)**
*P. aeruginosa* NCTC 6749	100 **(25)**	>800 (>800)	800 (400)	>800 (>800)	800 (400)
*P. aeruginosa* PAO1	100 **(25)**	>800 (>800)	800 (400)	>800 (>800)	800 (400)
*S. maltophilia* ATCC 13637	200 (100)	>800 **(12.5/25)**	100 (50)	>800 (800)	>800 (>800)
*S. maltophilia* ATCC 12714	400 (400)	>800 **(50)**	200 (100)	>800 (800)	>800 (>800)
*A. baumannii* ATCC 19606	>800 (800)	>800 **(400)**	200 **(50)**	>800 **(100)**	>800 (>800)
*B. cepacia* ATCC 25416	>800 (800)	>800 (>800)	800 (800)	>800 (>800)	800 (800)

alen, alendronate sodium; tic, ticlopidine; amit, amitriptyline; nic, nicergoline; crb, carboplatin; PAβN, Phe-Arg-β-naphthylamide. ^a^ When a difference between the minimum inhibitory concentration (MIC) values of the non-antibiotic active substance and medicinal product with and without PAβN was observed, both minimum inhibitory concentrations (MICs) of the active substance and its medicinal product are presented; ^b^ At least 4-fold decrease in the MIC of non-antibiotic active substances and medicinal products in the presence of PAβN, when compared with the MIC values of non-antibiotics without PAβN, is indicated in boldface.

**Table 2:** Susceptibility of clinical strains of Gram-negative rods to select quinolones in the presence or absence of PAβN.

Bacteria (No. of Isolates)	Quinolone ^a^	MICs Range (mg/L) ^b^		No. of Strains ^c^
		MH	MH + PAβN	
*E. coli* (*n* = 36)	**ofloxacin**	1–64	0.125–8	**36**
	nalidixic acid	1024 to >2048	64–256	36
*K. pneumoniae* (*n* = 36)	**ofloxacin**	4–32	0.25–4	**36**
	nalidixic acid	4 to >2048	1–512	36
*P. aeruginosa* (*n* = 36)	**ofloxacin**	2–128	0.063–4	**36**
	nalidixic acid	64–2048	1–32	36
*S. maltophilia* (*n* = 36)	ofloxacin	1–8	1–8	1
	**nalidixic acid**	4–32	1–4	**36**
*A. baumannii* (*n* = 36)	ofloxacin	8–64	4–64	5
	**nalidixic acid**	128–2048	32–256	**36**

PAβN, efflux pump inhibitor Phe-Arg-β-naphthylamide; MH, Mueller–Hinton II medium with 1 mM MgSO_4_. ^a^ Antimicrobial agents used to study the influence of non-antibiotics on antibacterial activity against different bacteria species are indicated in boldface; ^b^ The correctness of the assay was verified by determining the MIC of antimicrobial agents necessary to inhibit growth of reference strains (*E. coli* ATCC 25922 and *P. aeruginosa* ATCC 27853) and comparing with CLSI guidelines: ofloxacin MIC was 0.03 mg/L for *E. coli* and 2 mg/L for *P. aeruginosa*, nalidixic acid MIC was 2 mg/L for *E. coli* and 512 mg/L for *P. aeruginosa*; ^c^ Number of strains with at least 4-fold decrease in MICs in the presence of PAβN.

**Table 3:** The activity of non-antibiotics (active substances and medicinal products) against clinical isolates of Enterobacteriaceae and non-fermentative Gram-negative rods.

Bacteria (No. of Strains)	Non-Antibiotic Active Substance (Medicinal Product)	No. of Isolates with MIC Values			
		200 mg/L	400 mg/L	800 mg/L	>800 mg/L
*E. coli* (*n* = 36)	amitriptyline (Amitriptylinum)	7 (7)	27 (27)	2 (2)	0 (0)
*K. pneumoniae* (*n* = 36)	amitriptyline (Amitriptylinum)	23 (23)	13 (13)	0 (0)	0 (0)
*P. aeruginosa* (*n* = 36)	alendronate (Ostenil)	33 (33) ^a^	3 (3)	0 (0)	0 (0)
	amitriptyline (Amitriptylinum)	0 (0)	0 (0)	36 (36)	0 (0)
	carboplatin (Carbosin)	0 (0)	6 (6)	29 (29)	1 (1)
*S. maltophilia* (*n* = 36)	alendronate (Ostenil)	10 (10)	17 (16)	9 (10)	0 (0)
	amitriptyline (Amitriptylinum)	33 (33) ^a^	3 (3)	0 (0)	0 (0)
*A. baumannii* (*n* = 36)	amitriptyline (Amitriptylinum)	36 (36)	0 (0)	0 (0)	0 (0)

^a^ In the case of *P. aeruginosa* and *S. maltophilia*, for several strains (7 out of 33, and 2 out of 33, respectively) sensitivity to alendronate/Ostenil and amitriptyline/Amitriptylinum below 200 mg/L (MIC = 100 mg/L) was observed.

**Table 4:** Effects of PAβN on the MIC values of tested non-antibiotics against clinical isolates of Enterobacteriaceae and non-fermentative Gram-negative rods.

Bacteria (No. of Strains)	Non-Antibiotic Substance (Medicinal Product)	No. of Isolates with Indicated Fold Reduction in Non-Antibiotic MICs in the Presence of PAβN				
		≥4-Fold	≥8-Fold	≥16-Fold	≥32-Fold	≥64-Fold
*E. coli* (*n* = 36)	alendronate (Ostenil)	36 (36)	36 (35)	28 (26)	0 (0)	0 (0)
	ticlopidine (Apo-Clodin)	36 (36)	36 (36)	36 (36)	36 (36)	36 (36)
	amitriptyline (Amitriptylinum)	36 (36)	28 (28)	4 (4)	1 (1)	0 (0)
	nicergoline (Niglostin)	36 (36)	36 (36)	26 (25)	0 (0)	0 (0)
*K. pneumoniae* (*n* = 36)	alendronate (Ostenil)	30 (30)	19 (18)	0 (0)	0 (0)	0 (0)
	ticlopidine (Apo-Clodin)	36 (36)	36 (36)	36 (36)	33 (33)	31 (30)
	amitriptyline (Amitriptylinum)	27 (27)	2 (1)	1 (0)	0 (0)	0 (0)
	nicergoline (Niglostin)	30 (30)	28 (28)	7 (6)	0 (0)	0 (0)
*P. aeruginosa* (*n* = 36)	alendronate (Ostenil)	28 (28)	17 (16)	1 (1)	0 (0)	0 (0)
	amitriptyline (Amitriptylinum)	21 (21)	16 (15)	10 (10)	4 (3)	0 (0)
	carboplatin (Carbosin)	16 (16)	0 (0)	0 (0)	0 (0)	0 (0)
*S. maltophilia* (*n* = 36)	ticlopidine (Apo-Clodin)	33 (33)	21 (21)	16 (16)	13 (13)	4 (3)
*A. baumannii* (*n* = 36)	ticlopidine (Apo-Clodin)	30 (30)	27 (27)	24 (24)	21 (21)	8 (7)
	amitriptyline (Amitriptylinum)	24 (24)	3 (2)	1 (0)	0 (0)	0 (0)
	nicergoline (Niglostin)	36 (36)	36 (36)	11 (10)	1 (0)	0 (0)

PAβN, efflux pump inhibitor Phe-Arg-β-naphthylamide.
